# Sir Patrick Dun's Hospital

**Published:** 1831-04-01

**Authors:** 


### SIR PATRICK DUN'S HOSPITAL.

Cases of Diseased Brain. By Robert Law, M.D. Fellow of the King and Queen's College of Physicians in Ireland, &c.

[Dublin Hospital Reports, Vol. V.]

Case 1. *Tumour in the Brain.* A boy, aged 13 years, was admitted into Sir Patrick Dun's Hospital, for distressing palpitation of the heart, increased by quick walking, or ascending an eminence. His respiration was laborious and oppressed, and he was very plethoric. Before admission he had had two convulsive attacks, preceded by a dull pain in the left side of the head, from which he has never since been quite free. The treatment was exclusively directed to the cardiac affection. When six weeks in hospital, he was seized with an epileptic fit, followed by febrile symptoms, which degenerated into coma, in which state he died on the fourth day.

*Dissection.* "Considerable turgescence of the vessels on the surface of the brain. In the substance of the right hemisphere of the brain, and about an inch from the surface, there was a tumor as large as a hen's egg, hard and firm in its structure, while the brain in which it was imbedded was soft and diffluent; it was of a greenish yellow colour. The left ventricle was distended with limpid serum; the right ventricle contained no fluid.

The pericardium adhered to the heart through the medium of a dense adventitious membrane two lines in thickness, the heart larger than natural, lungs healthy, liver much increased in size, without any apparent deviation from its natural structure; mesenteric glands unusually developed. A section of the tumor exhibited a uniform consistence and colour."

The above case shews the difficulty of ascertaining organic diseases of the brain, and the imperfect measure which external symptoms afford of internal mischief!

*Case* 2. A girl, aged 12 years, became dull and languid, without making any complaint. Her face was shrunk, and countenance expressive of suffering. Her appetite declined, pulse became small and frequent—skin hot and dry—tongue loaded—breath offensive. And now she complained of flying pains in the abdomen, with confusion in her head on awaking in the morning. The abdomen was rather hard, but not tender. An evening exacerbation of fever took place at 7 o'clock—there was a teazing cough, with glairy expectoration. The respiration was feeble under each clavicle. A tumour formed on the scalp and suppurated—a similar one appeared on the right cheek—emaciation rapidly advanced—the fever increased—the abdominal pain became more severe—the skin harsh and dry. In the midst of these symptoms she was seized with violent pain in the head—her hands and arms became paralyzed—the countenance distorted, and death ensued in two days, the intellectual functions remaining undisturbed.

"*Dissection.* A circular portion of the cranium, corresponding to the suppurating tumor in the scalp, was denuded and rough, and immediately underneath it, and to the same extent, there was a deposition of coagulable lymph on the surface of the dura mater; on the surface of the brain, and in the ventricles, there was a considerable quantity of serum; the substance of the brain seemed as it were dissolved, and had the appearance of being soaked in water. In the left posterior lobe of the cerebrum, and not far from its surface, there was a tumor, the size of a nut, containing cheesy matter.

The apex of each lung was in a condensed state, and contained small encysted cheesy tubercles. The parietal and visceral peritoneum were so intimately united as to be separable only by dissection; also the convolutions of the intestines were firmly glued together by a thick adventitious membrane, in whose substance there were numberless cheesy tubercles, generally of the size of a pea. The liver was larger than natural, but exhibited no alteration of structure, it was only paler than usual: upon its convex surface, and corresponding to the attachment of the suspensory ligament, there was a tumor the size of a pigeon's egg, containing the same cheesy matter as the smaller tubercles. The mesenteric glands were not unusually large."

The above case exhibits a strong tendency to general formation of tubercles in the different cavities, with irritative fever. She was the tenth child of the same family, who had been carried off, one after another, by phthisis, and there can be little doubt that, had she survived the peritonitis, she would have fallen a victim to the family disease.

CASE 3. *Caries of Cranium—Mollescence of Brain.* A young female, aged 18 years, was delicate from infancy, and had suffered from glandular abscesses at the age of 7 or 8 years. Latterly several tumours formed on the head—softened, and ulcerated. She was then seized with convulsions, and became hemiplegiac of the right side. In this state she was admitted into hospital. There were now two ulcerations on the head, one of them laying bare the dura mater after destroying the bone. The heaving of the brain was here distinctly visible, and, at each elevation, a quantity of purulent matter was pumped up from under the edges of the sore. The muscular power of the right side was lost; but sensibility remained. The speech was almost unintelligible, and she complained of slight pain in the right side of the head. Leeches, purgatives, frictions, &c. relieved her so much that she left the hospital in six weeks much improved. She remained three weeks tolerably well—was then seized with fever—brought back to the hospital, and died in a few days, sometimes comatose, sometimes convulsed.

*Dissection* "In the situation of the ulcer above the left ear, a circular portion of the cranium, about eight lines in diameter, was entirely removed; the internal lamina of the left parietal bone for about a third of its extent was destroyed, exposing the cancellated structure of the diploe, there was also considerable caries of the inner plate of both parietal bones, and the frontal, where they meet at the superior fontanelles, as also in the course of the sagittal suture: the eroded surface was rough and irregular; there were numerous small circular perforations leading from the diseased internal portions of bone to a smaller diseased portion of the external lamina; they were of various dimensions, one was capable of admitting a large quill, while others did not exceed the small holes which afford passage to the nutrient vessels of the bone; through the larger one came the purulent matter which we observed to be pumped up at each motion of the brain. The externalor cranial surface of the dura mater corresponding to the diseased bone, presented a rough fungoid appearance, owing to a deposition of lymph, while the internal or cerebral surface was natural. The surface of the brain exhibited nothing peculiar, either in point of vascularity or colour, but in the substance of the left hemisphere and external to the corpus striatum there was a considerable breaking down and ramollissement of the cerebral structure, but no appearance of purulent matter; the lateral ventricles were distended with colourless serum.

The base of the left lung was in the first stage of pneumonia; all the abdominal viscera were healthy; the uterus was excessively small."

The above case is almost precisely similar to one detailed by Dr. Abercrombie, where the same destruction of bone, the same deposition of lymph on the dura mater, and the same disorganization of the cerebral substance obtained.

4. *Abscess of the Cerebellum.* A girl, aged eight years, was received into hospital with unequivocal signs of effusion in the head. She was quite comatose—the pupils widely dilated—pulse small and thready—extremities cold. She died in twelve hours after admission.

On dissection, there was found moderate congestion in the superficial veins of the brain. The substance was rather soft—the ventricles filled with serum—in the left lobe of the cerebellum a defined abscess, containing half an ounce of pus. The viscera of the thorax were healthy—the mesenteric glands enlarged.

5. *Hemiplegia, without evident Structural Changes in the Brain.* "John Stringer, aged 34 years, five years ago, while serving in the East India Company Service, received a bayonet wound in the forehead over the left eye, which penetrated the bone and gave rise to a sore which occasionally healed and broke out again; since the infliction of the wound he has never been quite free from uneasiness in his head; about six months since, while in bed, and without any previous warning, he was seized with paralysis of the right side and loss of speech; he partially recovered from these when he got the fever, for which he was admitted into hospital; he exhibited the following symptoms: quick pulse, tongue coated with yellowish fur, skin jaundiced and burning hot, epigastrium and right hypochondrium tender on pressure; a sore about the size of a six-pence over the left eyebrow, with exfoliation of the bone to such a degree as to expose the dura mater; complete paralysis of the right side with loss of speech; for a few days he shewed some signs of amendment, but suddenly be became insensible, in which state he continued until death.

*Dissection.* The portion of dura mater corresponding to the exfoliated bone seemed to have its fibrous lamina destroyed, while its serous lining was thickened and of a blueish colour; the subjacent brain had undergone no morbid change; there was considerable effusion on the surface of the brain. All the thoracic viscera were healthy; the liver enormously enlarged, weighed ten pounds, but did not appear to have its structure altered; the spleen, nearly three times its natural size, exhibited a strange appearance; its superior third was converted into a yellowish, soft, cheesy substance, the middle third had undergone that breaking down, that softened condition, which prolonged ague often produces in this organ; the inferior third had retained its natural organization and consistence; the mucous membrane of the stomach was deeply vascular."

6. *Hemiplegia, with Ramollissement of Brain.* The subject of this case was a watchman, of irregular habits, who a week previously was apparently in the enjoyment of health. He was seized with apoplexy, and lay in a state of stupor, for some time; but was at length roused by copious bleeding. His left side was then paralytic, and his speech lost. In this state he was carried to the hospital, where he continued for two months, with a low typhoid fever, under which he at length sank.

On dissection, there was found extensive softening of the thalami optici and corpora striata.

The two last cases are placed in juxta-position by our author, to shew that opposite conditions of the brain will give rise to the same symptoms during life! This is a mortifying truth —but it cannot be concealed! It shews, as the daily experience of every man shews, that medicine never can approach to any thing like an exact science! We must grope in the dark till the end of time. Experience alone can confer on men—and not on all men—the art of groping, with some chance of finding their way!

II. Hæmatemesis dependent on Disease of the Liver. By R. Law, M.B.

[Dub. Med. Trans.]

*Case* 1. Mary Treyne, aged 43, was seized suddenly, four days ago, with vomiting of blood, and discharges from the bowels, of similar kind, continued till she entered the hospital. On the day of admission she vomitted not less than a quart of coagulated blood, and exhibited all the symptoms characteristic of such a loss. Infusion of roses with sulphate of magnesia and sulph. acid was administered, and hot water was applied to the lower extremities. 28th Nov. vomited very little blood, but had frequent discharges from the bowels. She died in the course of the evening.

On examination, the lungs were found to be quite healthy—heart soft, flabby, and pale—stomach containing a pint of blood, and the intestines containing much of the black tarry matter such as had been passed by stool. "The entire tract of the gastro-intestinal mucous membrane so far from exhibiting any unusual vascularity, seemed quite blanched."

"The liver presented an irregular tuberculated or granulated surface; was contracted in size; its anterior margin much less acute than natural. A section of it exhibited small round bodies of various dimensions, separated by dense fibro-cellular septa; this fibro-cellular tissue seemed to be the proper cellular tissue of the organ increased in density, furnishing loculi or capsules to these roundish bodies, which are probably the acini in a state of hypertrophy; these bodies adhered loosely to their capsules, and could be easily detached from them; the consistence of the organ was greater than natural; its colour a whitish grey."

*Case* 2. Peter Quinn, aged 38, a hotel waiter, was admitted in Nov. 1827, for vomiting of blood, which was soon followed by ascites. This last yielded to mercurial medicines and vegetable diuretics. He was discharged cured in January, continuing well till the ensuing September, when he was again seized with vomiting of blood, and with dropsical swellings, not only of the abdomen, but also of the lower extremities and the scrotum. His breathing was short and laboured—the surface of the body jaundiced. No medicine made any impression on the complaint. Paracentesis abdominis was then performed —peritoneal inflammation ensued—and he died in thirty-six hours.

The lungs were healthy—the heart small and flabby. The peritoneum was highly inflamed, and the mucous membrane of the stomach was injected, while that of the intestines was pale. The liver presented the same irregularity of surface and deviation from natural structure as in the preceding case. The author met with two other cases almost exactly similar, and where the appearances after death were the same.

"These cases unequivocally prove the vomiting of blood to depend upon the peculiar alteration of structure of the liver, which was found to exist in all; and it is remarkable, that although almost every pathologist has noticed this deviation from natural structure in the liver; Frank seems to be the only one who has observed how often it and hæmatemesis stand to each other, in the relation of cause and effect.

This diseased condition of the liver would seem to consist in an entire change of the proper substance of the organ, into a dense fibro-cellular tissue, forming a kind of pulp or parenchyma, in which the roundish bodies (which projecting on the surface, give the organ its irregular appearance) are imbedded, its specific weight is greater than natural, while its actual size is less, and so far from passing the margin of the ribs, it seems to have receded within its ordinary bounds, showing how fallible a criterion of the health of the organ, is either its size or its descent below the ribs; its form is altered; it becomes more round; its anterior margin more obtuse; its division into lobes less defined; its peritoneal covering thicker and more opaque than natural; its section exhibits no trace of blood vessels, which we may presume to be either compressed or obliterated by the altered structure; hence the impediment to the vena portæ pouring its blood into its ordinary channels, and the influence of the obstruction reverts upon all the branches which concur to form this vein, and thus we do satisfactorily account for the hæmorrhage; this fact would tend to confirm an opinion of the ancients respecting the office of the liver; that it was a diverticulum or reservoir of the abdominal venous circulation. As this pathological condition of the liver is most frequently met with in persons who have indulged freely in the use of ardent spirits, and as we know that the direct tendency of such an indulgence is to produce a chronic inflammation of the gastro-intestinal mucous membrane, would it be pushing conjecture too far to suppose the disease of the liver to depend upon the extension of inflammation, from the intestine along the ductus communis choledochus?"

### III. CASES OF PUTREFACTIVE DISORGANIZATION OF THE LUNGS. By ROBERT LAW, A. M. &c.

[Dublin Med. Trans.]

Modern pathologists have applied the term "gangrene" to a diseased condition of the lungs, one peculiar character of which is the fetid expectoration. The disease in question will not bear comparison with gangrene as it occurs on external parts, and as cognizable to the senses.

"This modification of disease, unlike gangrene occurring as a termination of inflammation, never exhibits, at any period of its progress, any of the legitimate characters either constitutional or local, of genuine inflammation; it develops itself in a constitution either naturally weak, or rendered so by previous disease, and such as we should, a priori, say would be unequal to the effort of healthy inflammation; the internal mischief is not announced either by an acceleration of pulse or heat of skin. The patient first complains of a sharp pain in the side, which is soon succeeded by a profuse discharge of either pure blood, or of a mixture of blood and purulent matter; he now feels himself much lighter, relieved as it were from a load, and enjoys an interval of ease, when he renews his complaint of his breathing becoming much oppressed; he is again relieved by discharging a large quantity of mixed blood and pus; he thus continues for some time alternating between comparative ease and distress, till a teasing cough with a fetid expectoration establishes itself, the sputa are generally of a greenish purulent character, floating in abundant thin mucus, and sometimes so acrid as to create a burning sensation in the throat; the breath emits a most offensive fœtor; the complexion is either a dusky yellow or a dead white, and always expresses distress and suffering. The sallowness of the complexion which I had observed in two cases, led me to suspect that the liver was involved; however, examination after death did not confirm my suspicions, though in both cases the disease was confined to the right side. The constitution soon begins to sympathise, and hectic fever sets in, but by no means observes the regularity and uniformity of the hectic which accompanies phthisis pulmonalis. The emaciation proceeds; the appetite fails; nothing can allay the irritation of the cough; the fœtor of the expectoration is insupportable both to the patient and the attendants; the bowels become much deranged. If you inspect the chest you will observe, in general, the part corresponding to the affected portion of the lung quite motionless, as if it took no part in the act of respiration. Nothing is more uncertain than the duration of this disease, before it arrives at its fatal termination. I have had one case under my care for more than a year, and have known another which survived the first attack more than a year and half; if it be combined with tubercular phthisis, it runs a more rapid course, and has its character modified by the complication; in general, death seems to be owing to the irritation of the system, rather than to the extent of the local mischief. The anatomic characters of the disease identify it with Hunter's description of unhealthy suppuration or with what Travers describes as the results of Gangrenous inflammation, or inflammation whose powers are inadequate to carry it to a healthy termination; these results are imperfect adhesion, an unhealthy suppuration, and a vitiated effusion at the expense of the life of the parts, complicated with a chemical decomposition of both fluids and solids."

We now proceed to the cases, which we shall considerably abridge.

*Case* 1. J. Dunne, aged 19, a thin delicate boy, was admitted into Sir P. Dun's Hospital, for fever, in the progress of which he was seized with a profuse expectoration of "fluid arterial blood,"* which he said had often happened before. Camphor, digitalis, and opium were administered. The hæmoptysis ceased. After various turns of illness he at length left the hospital; but returned again, decidedly hectic, his breath and expectoration being extremely fetid. In all the right lung respiration could scarcely be heard—under the left clavicle was imperfect pectoriloquy. He died in two days from this time, and six weeks after admission into hospital.

*Dissection.* "Head not examined; the apex of the right lung adhered so firmly to the corresponding part of the cavity of the chest, that it could not be separated without breaking its structure; this lung was much heavier than natural, and felt quite solid, except at its base; the investing pleura was universally thickened, and in some places had acquired the density of fibrocartilage. The entire substance of the lung, except the base, was thickly studded with tubercles; the pulmonary tissue surrounding these bodies was either broken down into a soft brownish sloughy substance, or so condensed, as to have its cellular nature quite destroyed; there were many irregular cavities traversed by bands of pulmonary structure; the surface of each cavity exhibited a blackish sloughy appearance; the base of the lung was quite free from tubercles, but was in the first stage of pneumonia.

The left lung exhibited a similarly disorganized condition, the small irregular cavities were more numerous, and the intervening pulmonary structure softer, presenting the same dirty sloughy broken down appearance. On pursuing some of the bronchial ramifications which opened into these cavities, their lining membrane was highly vascular, and, in some instances, black; the base of this lung, too, was in the first stage of pneumonia, and free from tubercles; the left cavity of the pleura contained about a pint of straw-coloured serum; the pericardium about eight ounces of the same fluid. There was also an infiltration into the subserous tissue, connecting the substance of the heart and the pericardium; the heart was small and flabby; the abdomen contained about two quarts of serum, all the viscera of this cavity were healthy."

*Case* 2. Arthur Maguire, aged 37, in embarking for England fell into the river, and continued in his wet clothes till the vessel reached Liverpool. Five days afterwards he was seized with pleuritic stitches in the left side, and soon began to spit blood and matter, in consequence of which he returned home, and came into hospital. On the day of admission, he discharged about a quart of "fluid arterial blood"—his countenance was pale and exsanguious—pulse feeble. He referred the pain to the left side

* Dr. Law must be aware that *arterial* blood from the lungs is *black* blood.—*Ed.*

below the nipple. Pectoriloquy with bruit amphorique was distinctly heard in this point. This side was distinctly more prominent than the right; and, on percussion, yielded a dull sound posteriorly and inferiorly, where respiration was inaudible. The diagnosis marked was—"cavity communicating with the cavity of the pleura, and effusion into the left side." The spitting of blood ceased; but was succeeded by cough and purulent expectoration, which soon became so fetid, that it was disagreeable to approach him. At intervals he discharged pints of greenish matter, which sometimes resembled, in colour and consistence, the yolk of egg. His breathing was relieved by these discharges. Obstinate diarrhœa, with colliquative perspirations set in, and he died in seven weeks from his admission into hospital.

*Dissection, 24 hours after death.*—"On opening the thorax, a considerable quantity of air escaped from the left side; there was an effusion to the extent of two pints of thin greenish purulent fluid into the left cavity of the chest; a dense coating of lymph connected both pleura pulmonalis and costalis on this side in all points, except along the side of the spine, where these two membranes firmly adhered; the lung was compressed to about two-thirds of its natural size; the superior half was quite free from disease; in the middle of the organ, and about midway between the anterior and posterior margins, there was a cavity capable of holding a hen's egg, not lined with any defined membrane, but by the unequal jagged sloughy tissue of the lung; this cavity opened into the cavity of the pleura by an irregular opening about the size of a half crown piece. Into the large cavity several smaller ones opened, all presenting the same unequal sloughy appearance. The base of the lung was hepatized, the bronchial glands were much enlarged, and contained an inky fluid in their centre; the right lung was quite free from disease. This case resembled pneumonia in its anatomic characters more than phthisis, but in its constitutional symptoms, at no period did it evince the high inflammatory action of true pneumonia."

*Case* 3. John Walsh, aged 41, was seized, after exposure to cold, with pain under his right breast, and in ten days afterwards had cough and spitting. At the end of the fifth week he discharged a pint of purulent matter. From this time, cough with fetid expectoration, were the principal phenomena. The disease gradually sapped the foundations of life, and death put an end to his sufferings. Before this event the right side of the chest became quite motionless. It did not seem to act in respiration.

*Examination.* "The body very much emaciated; the right pleura pulmonalis and costalis adhered so firmly, that with great difficulty could they be separated; the lung was much heavier than natural. On making a section of it from its apex to its base, there were innumerable small cavities with sloughy broken surfaces; the general structure of the organ resembled the dirty greenish softened pulp, to which we sometimes find the spleen reduced; a thin blackish matter exuded, emitting a most disgusting fœtor."

*Case* 4. John King, aged 24, of sallow and unhealthy appearance, stated that, about a month previously, after exposure to cold in a fit of intoxication, he was seized with severe pain in the right side, which was soon followed by cough, profuse spitting of blood, and difficulty of breathing. After a week he applied to a dispensary, where he was bled, blistered, and physicked, with some relief:—but he still continued to expectorate large quantities of blood, and also passed blood by stool. On the 22d Jan. the following symptoms were noted.

"Countenance of a pale leaden hue; lips white and bloodless; pulse 120; respiration weak; very much hurried; great prostration of strength; appetite quite gone; excessive thirst; cough very teazing, coming on in paroxysms; breath most offensive; sputa have the appearance and smell of putrid blood; he sometimes discharges nearly a pint of this blood at a time, and seems to vomit rather than expectorate it; from which, and from his discharging it by the bowels, I fancy he swallowed it in the fits of coughing; is constantly bathed in cold clammy perspiration. Percussion and auscultation bespeak the left lung healthy; the right side of the chest sounds dull, in its superior two-thirds both anteriorly and posteriorly. Gargouillment, cavernous respiration, and imperfect pectoriloquy heard to the same extent anteriorly."

He died four hours after admission into hospital.

"Body not much emaciated; almost the entire of the right lung was hollowed out into a cavity, containing a large quantity of grumous blood in a state of putrefaction; this cavity was lined by a membrane, the small remains of pulmonary structure in its immediate neighbourhood had undergone imperfect hepatization, and resembled liver that had been macerated; the left lung quite healthy; the pleura investing its base, and also, the corresponding portion of this membrane lining the diaphragm, were converted into fibro-cartilage; the heart was small and flabby."

This case convinced our author of a circumstance which he had long suspected — that profuse discharges of blood in cases where we know the lungs to be affected, often come from the stomach, in consequence of the patient swallowing the blood that comes from the lungs, and which, when accumulated, excites vomiting.

This paper of Dr. Law's concludes with a long critical note on Dr. W. Philip's doctrine respecting dyspeptic phthisis. From this note we shall only quote one short passage, and our readers will acknowledge that we have often made the same remark which Dr. Law makes.

"When Dr. Philip observes, it is surprising the state from which he has seen the lungs recover on the removal of hepatic irritation, I suspect, had he employed auscultation, he would have ascertained the integrity of the organ, and that this state of the lungs consisted in mere functional disorder. I cannot help questioning the accuracy of Dr. Philip's views on this subject, when I find him asserting that he has seen a person in the last stage of phthisis saved, by the glands of the neck swelling and suppurating."

Scarcely a day passes in which we do not see patients pronounced to be labouring under "*dyspeptic phthisis*," and where the whole attention is directed to the liver and digestive organs, when, in reality, the lungs are found to be in a state of complete disorganization. If auscultation do no other good than dissipate this hallucination respecting "dyspeptic phthisis," the labours of Laennec deserve a crown of glory.

### IV. REMARKABLE CASE OF OVARIAN DISEASE. By W. F. MONTGOMERY, A.M. &c.*

Mary Clarke, ætatis 45, mother of nine children, the youngest nine years old, was admitted under Dr. Montgomery into Sir Patrick Dun's Hospital, on the 13th Aug. 1828. There was slight ascites, and a tumour in the right iliac region. There were frequent discharges from the vagina of a fluid like water, and, on examination, a small cauliflower excrescence of the os uteri was detected; the urine was scanty, the pulse weak, and the countenance expressive of peculiar distress. She had first perceived the tumour ten years previously; the ascites had commenced within the last four months. She had formerly had hernia on both sides, and the sac on the right was now distended with the dropsical fluid.

Diuretics were prescribed; but on the 17th, she complained of pain in the abdomen, relieved by leeches and the hip-bath. The secretion of urine was greatly increased, but so was the ascites, and the uneasiness was such, that on the 31st she was tapped by Dr. Jacob at the left side, as far as possible from the tumour. Six quarts of clear fluid were drawn off, with great relief, and, along with the fluid, several thin membranous flakes passed through the

* Dublin Med. Transactions, Vol. I. Part I.

canula, and were thought by Dr. Montgomery to be ruptured hydatids. The abdominal tumour could now be traced down into the pelvis at the right side, and extended beyond the median line of the abdomen. In four days, a return of the ascites was taking place, with anasarca of the left leg and thigh. We need not specify the means employed; they procured no benefit. On the 11th Sept. the operation of tapping was again performed, with the same relief as before, a substance of a reddish colour and gelatinous consistence escaping through the canula. The tumour was now at least half as large again as on the first tapping, twelve days previously. Diarrhœa was now established—she rapidly sank, and on the 21st she died. The water had not re-accumulated so rapidly after the last tapping.

*Sectio Cadaveris*. Great emaciation; left leg and thigh very anasarcous. In the abdomen, much deep yellow-coloured serum, and at least two pints of pus.

"On turning aside the integuments, a very singular appearance presented itself; a tumour chiefly composed of fine membranes, dividing it into innumerable cells, which, with their fluid and transparent contents, resembled, at first sight, hydatids; the membranous septa dividing the cells were supplied with blood-vessels of a considerable size running along their edges, so that the whole tumour presented a clear red colour. At its upper and left part there was a deep cleft or fissure, into which the open hand might be passed without any force, and when carried downwards, and towards the right side, it entered a round sac equal in size, and much resembling a large flat turnip; this was the right ovary which lay just under, and was filled with the same structure as the part of the tumour first brought into view.

In fact, it seemed as if the peculiar structure had at first grown in the ovary, which thereby became greatly enlarged, until at length the coat of the ovary had given way, and out of the fissure so formed, the morbid growth continued to enlarge, turning over the edges of the fissure, and covering the front and sides of the ovary in which *it had formerly been contained*, so that the tumour was in a great measure turned inside out.

This change in the state of the tumour might, I conceive, have happened in one of two ways; either by the coat of the ovary giving way to the pressure of the morbid growth within it, which seems probable, from the circumstance of that substance having evidently continued to grow out of the fissure; or the breach in the coat or capsule of the ovary might have been produced by external violence or accident, a cause but too probably true, as I afterwards ascertained that the poor creature had been exposed to a great deal of ill-treatment from a brutal husband. The tumour was of such a size, that while its inferior extremity was in the pelvis, its superior border was as high as the ensiform cartilage, its length being twelve inches and its breadth nine."

The tumour adhered to the neighbouring parts by some slight, but firm, membranous bands. The uterus was twice its ordinary size, and scirrhous; the os uteri exhibited the numerous flocculent processes, which are all that remain of cauliflower excrescence after death. The left ovary was somewhat enlarged, tuberculated on its surface, and very hard.

"On this case and dissection I would now wish to make two or three brief observations. With regard to the tumour itself, without wishing to theorize or offer any opinion on its peculiar nature, I believe, in the first place, that it is a form of disease not before observed, as affecting the ovary, or at least not hitherto described, as far as my research enables me to speak. I have dissected a great number of cases of ovarian disease, and have preserved specimens in my museum, of almost all the different species enumerated by authors, but to none of these does the disease in this case bear the slightest resemblance in character; another peculiarity, which I look upon as very remarkable, consists in the open state of the tumour, and its internal surface being in consequence exposed in the living body, and literally in a great degree turned inside out.

From the surface thus exposed, serum must have been abundantly poured out, and hence, perhaps a cause, or at least one source of the effusion into the peritoneum, and whether the circumstance can be fairly attributed to this, or to some other more general cause, it is to be recollected, that during the period in which the effusion took place most rapidly, the tumour was found to have nearly doubled its surface.

The state of the tumour appears to me also to account for the great disproportion between the uneasiness felt and the degree of distention existing.

It seems not so easy to account for the œdema of the thigh and leg occurring at the left side, while the tumour was at the right. A very intelligent pupil, Mr. Dwyer, who gave me his valuable assistance in the dissection, suggested that it might perhaps have been caused by the weight of the tumour pushing the enlarged and scirrhous uterus forcibly to the opposite side of the pelvis, and I agree in the probable correctness of this ingenious suggestion."

---

